# Evolution of CD8^+^ T Cell Receptor (TCR) Engineered Therapies for the Treatment of Cancer

**DOI:** 10.3390/cells10092379

**Published:** 2021-09-10

**Authors:** Yimo Sun, Fenge Li, Heather Sonnemann, Kyle R. Jackson, Amjad H. Talukder, Arjun S. Katailiha, Gregory Lizee

**Affiliations:** 1 Department of Melanoma, University of Texas M.D. Anderson Cancer Center, Houston, TX 77030, USA; YSun16@mdanderson.org (Y.S.); fli3@mdanderson.org (F.L.); HMSonnemann@mdanderson.org (H.S.); KRJackson1@mdanderson.org (K.R.J.); atalukde@mdanderson.org (A.H.T.); ASKatailiha@mdanderson.org (A.S.K.); 2 Department of Immunology, University of Texas M.D. Anderson Cancer Center, Houston, TX 77030, USA

**Keywords:** T-cell receptor (TCR), engineered TCR-T cell therapy, tumor antigen, clinical trials, cancer treatment

## Abstract

Engineered T cell receptor T (TCR-T) cell therapy has facilitated the generation of increasingly reliable tumor antigen-specific adaptable cellular products for the treatment of human cancer. TCR-T cell therapies were initially focused on targeting shared tumor-associated peptide targets, including melanoma differentiation and cancer-testis antigens. With recent technological developments, it has become feasible to target neoantigens derived from tumor somatic mutations, which represents a highly personalized therapy, since most neoantigens are patient-specific and are rarely shared between patients. TCR-T therapies have been tested for clinical efficacy in treating solid tumors in many preclinical studies and clinical trials all over the world. However, the efficacy of TCR-T therapy for the treatment of solid tumors has been limited by a number of factors, including low TCR avidity, off-target toxicities, and target antigen loss leading to tumor escape. In this review, we discuss the process of deriving tumor antigen-specific TCRs, including the identification of appropriate tumor antigen targets, expansion of antigen-specific T cells, and TCR cloning and validation, including techniques and tools for TCR-T cell vector construction and expression. We highlight the achievements of recent clinical trials of engineered TCR-T cell therapies and discuss the current challenges and potential solutions for improving their safety and efficacy, insights that may help guide future TCR-T studies in cancer.

## 1. Introduction

Adoptive T cell transfer (ACT) is one of the most promising immunotherapy approaches in cancer treatment. There are currently four well-developed ACT techniques, including autologous tumor-infiltrating lymphocyte (TIL) therapy, antigen-specific endogenous T-cell therapy (ETC), T-cell receptor engineered T cell therapy (TCR-T), and chimeric antigen receptor T-cell therapy (CAR-T). While TIL and ETC therapies rely on the isolation and in vitro expansion of T cells derived from tumor or peripheral blood, respectively, TCR-T and CAR-T therapies use genetic modification of T lymphocytes to endow them with tumor antigen specificity [1].

Genetic modification of lymphocytes was first proposed over 30 years ago, and there have been many developments in the field since then that have facilitated their clinical utility. As discussed elsewhere in this issue, CAR-T approaches have been highly successful at treating certain hematopoietic cancers, particularly those directed against the B cell-specific surface protein CD19. CAR-T therapies use single chain variable fragment (scFv) chimeric antigen receptors (CAR) that can directly recognize tumor cell surface antigens without the requirement for MHC restriction, which is one major limitation of TCR-directed immunotherapies [2]. These CAR constructs can also incorporate the intracellular domains of several different co-stimulatory molecules, which can facilitate T cells’ activation, proliferation, and effector functions. However, while CAR-T therapies are highly promising, they are limited to targeting tumor cell surface proteins that usually do not show a high degree of tumor specificity and have demonstrated little clinical efficacy in the setting of solid tumors. As discussed further below, TCR-T cell approaches can overcome both of these issues and have produced compelling clinical data in solid cancers. While CARs use an antibody-like structure for antigen recognition, TCR-T cells utilize a full TCR complex, which includes a heterodimer consisting of TCR α- and β-chains, with T-cell signaling upon antigen recognition conveyed naturally by clusters of CD3 chains [3]. With expression of the complete TCR complex, engineered TCR-T cells can recognize polypeptide fragments expressed both within the tumor cell and at the cell surface, thus offering a much broader range of target antigens. Historically, TCR-T targets have included carcinoembryonic antigen (CEA, colorectal cancer), glycoprotein gp100 (PMEL, melanoma), melanoma antigen recognized by T-cells 1 (MART-1, melanoma), melanoma-associated antigen 3 (MAGE-A3) (melanoma/multiple myeloma), and New York esophageal squamous cell carcinoma 1 (NY-ESO-1) (melanoma/synovial cell sarcoma) [4]. More recently, TCR-T cells have also targeted neoantigens generated by somatic mutations in tumor DNA, which are more tumor-specific but are also less shared by cancer patients [5].

These engineering approaches can successfully redirect T cells’ specificity to selectively target tumor-associated antigens. However, several significant limitations of these approaches remain to be overcome. Here, we review the current technologies and workflows for producing TCR-T cell therapeutics. We summarize the tumor antigens currently being targeted with engineered TCR-T therapies and evaluate the clinical efficacy of current clinical trials. Also, we outline the challenges that need to be addressed in order to improve the success of TCR-T approaches and discuss potential resolutions. Lastly, we provide perspectives on the key goals and objectives for future research, which include the personalized identification of therapeutic tumor-specific TCRs and potential modifications to improve TCR signaling and effector function.

## 2. Definition and Mechanistic Overview of TCR-T Cell Immunotherapy

**Figure 1:**
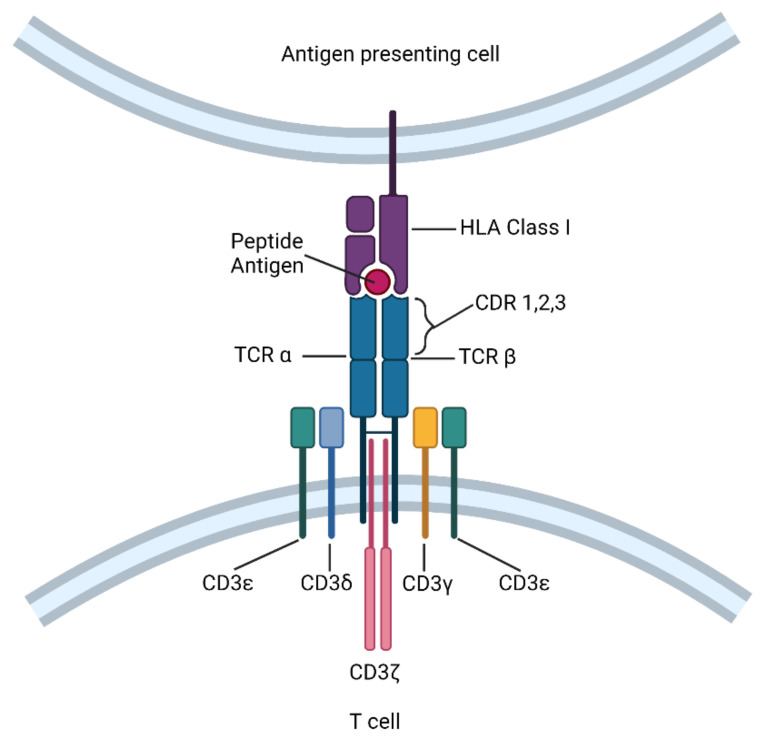
The T cell receptor (TCR) structure and TCR-T cell components. The TCR comprises an α chain and a β chain. TCR-T is a T cell engineered with an antigen-specific TCR.

Cytotoxic CD8^+^ T cells recognize foreign antigens on target cells in the form of peptides bound to human leukocyte antigen Class I molecules (HLA-I) through a heterodimeric TCR consisting of α- and β-chains [6]. Following recognition of cognate antigen, information about the quantity and quality of antigen engagement is relayed through intracellular signal transduction, facilitating T-cell effector function. While TCR itself lacks a significant intracellular domain, it is associated with invariant CD3 dimers—CD3γε, CD3δε, and CD3ζζ—to activate the downstream signaling machinery (Figure 1) [7]. The CD3 ε, γ, δ, and ζ chains each contain one (ε, γ, and δ) or three (ζ) immunoreceptor tyrosine-based activation motifs (ITAMs) that undergo phosphorylation by the Src kinase Lck, thereby initiating a downstream T cell signaling cascade [8]. In this way, the TCR-CD3 complex bridges the signaling between HLA-I/peptide recognition and T cell activation.

TCRs that engage with HLA-I/peptide complexes contain three complementarity-determining region (CDR) loops on each of the Vα and Vβ domains [9]. Among these, CDR1 and CDR2 are encoded within the TCR’s variable segments, whereas CDR3 is formed by a DNA recombination event involving the adjacent Vα and Jα segments for α chain genes and Vβ, D, and Jβ segments for β chain genes. By constructing TCR α and β chains from CDRs with a defined antigen specificity, ectopically expressed TCRs can utilize the CD3 complex endogenous to T cells. This method was used in transgenic mice to demonstrate that a transgenic TCR was sufficient to direct antigen-specific T cell differentiation, and was later applied to human T cell clones to redirect their cytotoxicity [10,11]. Because TCR-T cells retain all the auxiliary molecules of the TCR signal transduction pathway, these cells can become fully activated at low target cell antigen densities, and may potentially outperform CAR-T cell recognition [5,12]. In addition, TCR-T cell infusions have resulted in fewer adverse events, including acute cytokine release syndrome and neurotoxicity [13], potentially due to the autoregulatory capacity of the natural TCR-CD3 complex. However, since TCR-T cells recognize specific HLA-I/peptide complexes, their use is limited to only those patients who express the appropriate HLA-I allotype.

## 3. Tools and Techniques for TCR-T Cell Development

**Figure 2:**
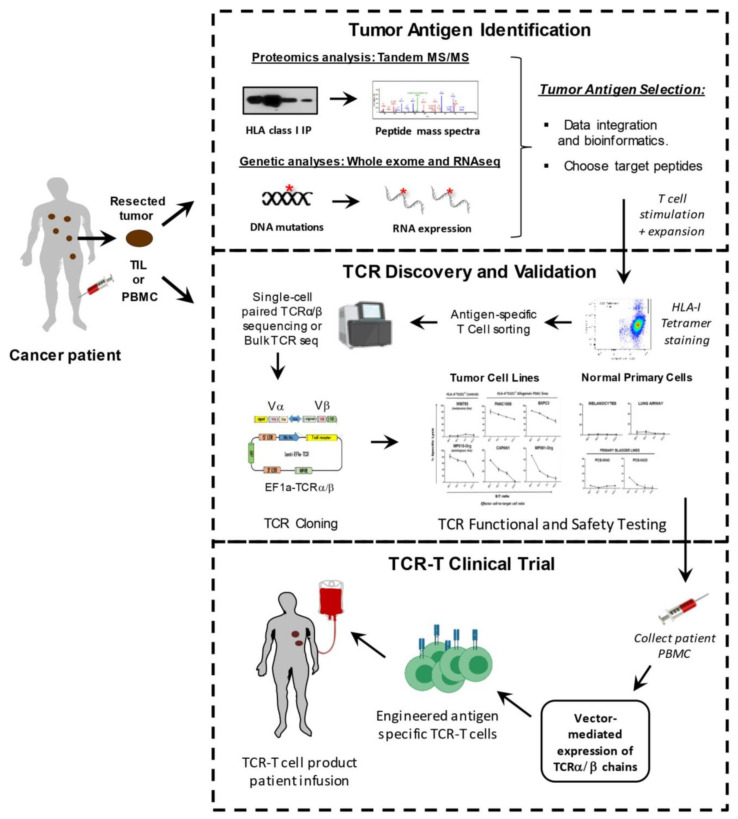
Workflow of antigen discovery, TCR cloning, and TCR-T reconstruction. Development of TCR-T immunotherapy can be facilitated by three separate working modules: tumor antigen identification, TCR discovery and validation, and TCR-T therapy clinical trials.

To isolate therapeutic TCRs, antigen-specific T cells must first be isolated from the blood of patients or healthy donors, and expanded in vitro with specific peptide antigen along with γ-chain cytokines including IL-2, IL-7, IL-15, and IL-21 [12]. This process requires the prior identification of specific tumor-associated peptide targets that can be targeted safely in patients. After the target antigen has been selected, different methods can be used to screen TCRs that have the desired high affinity and tumor specificity. Preclinical safety testing is also necessary to ensure minimal off-target effects and cross-reactivity of isolated high-affinity TCRs. Viral vectors are often used to genetically modify autologous patient T cells to express validated, therapeutic TCRs, prior to being transfused back into the patient [4] (Figure 2).

### 3.1. Target Antigens for TCR-T Therapy

Melanoma antigen recognized by T-cells 1 (MART-1) was the first tumor-associated antigen targeted in a TCR-T clinical trial, as reported by Steven Rosenberg’s group in 2006 [14]. Adoptive transfer of MART-1-specific TCR-T cells in 15 patients resulted in durable engraftment at levels exceeding 10% of peripheral blood lymphocytes for at least 2 months after the infusion and showed beneficial effects, including tumor regression [15]. In addition to anti-tumor effects, several patients also showed on-target toxicities against normal melanocytes, leading to vision or hearing problems, but these were largely resolved with steroid treatment. Following this breakthrough, TCR-T therapies against a wide range of tumor antigens have been developed, including those targeting MAGE-A3, MAGE-A4, GD2, mesothelin, gp100, MART1, AFP, CEA, NY-ESO-1, and viral peptides derived from HPV and EBV [16,17,18]. Among these, NY-ESO-1 has been shown to be one of the most promising targets for TCR-T cells, achieving success in treating synovial sarcoma with an objective response rate of 67% [17,19] (Table 1).

Ideal TCR-T target antigens are those expressed exclusively on tumor cells to avoid toxicities against normal tissues. In addition, ideal targets display the following features: (1) the capacity to elicit an immune response (e.g., immunogenicity), (2) associated with driving the tumor phenotype (e.g., oncogenes) to reduce the risk of antigen loss and tumor immune evasion, and (3) expression on cancer stem cells to promote permanent tumor eradication [20]. Neoantigens (NeoAg) are peptide targets derived from somatic DNA alterations and demonstrate many of the features considered ideal. However, the wide diversity of somatic mutational profiles, combined with high HLA-I polymorphism, means that the vast majority of tumor neoantigens are personalized and not shared among cancer patients. As discussed further below, they are most often identified through predictive methods and not validated through empirical means, with a few important exceptions. Tumor-associated antigens (TAA) are peptides that originate from endogenous wild-type proteins whose expression is elevated in tumors but limited in magnitude or in spatial expression in healthy tissues. While they do not demonstrate the absolute tumor specificity of NeoAgs, TAAs have the advantages of being widely shared among cancer patients and being considerably easier to identify and validate [1].

### 3.2. Methods for Identifying Tumor-Associated Antigens

High-resolution mass spectrometry (MS) has been shown to be the most robust high-throughput approach for facilitating the direct identification of HLA-I bound peptides from tumor cells [21,22,23]. By this method, HLA-I/peptide complexes are isolated from tumor tissues or cell lines by immunoprecipitation (IP), followed by extensive washing and application of an acidic elution buffer to dissociate the bound peptide antigens from the HLA-I molecules and the antibody used for IP [24]. This strategy has allowed the identification of thousands of validated peptide targets per tumor sample, and has been used to identify HLA-I ligandomes for glioblastoma (GB), melanoma, renal cell cancer (RCC), and colorectal cancer (CRC), among others [24,25,26]. Our group has established a high-throughput MS-based pipeline together with RNA sequencing to identify tumor-associated antigens from individual cancer patients. Using this method, we have identified and validated peptides associated with the melanoma differentiation antigen SLC45A2 as shared melanoma-associated tumor targets and have generated TCRs recognizing these peptides. Similarly, we identified VGLL1 as a shared pancreatic and basal breast cancer tumor-associated antigen, for which TCRs showing antitumor activity have been isolated [27,28,29]. TCR-T cell clinical trials targeting these two TAAs are currently in the design stages.

### 3.3. Approaches for Identifying Tumor Neoantigens

Although MS-based techniques can be used to identify neoantigens, they are much more refractory to identification due to their relatively low abundance combined with the limited sensitivity of MS, particularly with tumor samples of limited size. However, the development of next-generation sequencing techniques has been instrumental in identifying and targeting this class of tumor targets. Whole exome DNA sequencing, combined with computation prediction algorithms, allows for the identification of particular genetic alterations in cancer cells that can generate mutated peptides with the capacity to be presented on tumor HLA-I molecules [30]. All somatically-mutated genes can be subjected to in silico analysis to predict potential high-affinity epitopes that may bind to the patient’s individual HLA-I molecules and thus be potentially recognized by T cells [31,32,33]. HLA-I peptide binding prediction algorithms are constantly being updated and improved with the use of large MS-eluted peptide databases, and other prediction algorithms attempt to take into consideration biological variables related to the complexity of the intracellular processes governing peptide fragmentation by the proteasome and the transport of peptides to HLA Class I molecules in the endoplasmic reticulum [34]. Other selection filters may be applied to eliminate peptides predicted to be poorly processed by the immunoproteasome or that have lower binding affinity than the corresponding wild-type sequences [35]. An additional parameter often applied includes tumor RNA sequencing, which allows for selection of the putative NeoAgs with the highest transcript expression, which are more likely to produce abundant peptides for presentation [36]. It is important to note that while these prediction methods can often show very good accuracy in identifying presented and/or immunogenic NeoAgs, they typically predict numbers of putative NeoAg targets that are 1 to 2 logs higher than the actual number of bona fide targets.

Neoantigen discovery via trogocytosis is a novel approach that has emerged in recent years. Trogocytosis is a biological phenomenon that happens during cell conjugation. During this process, cells share and transfer membranes and membrane-associated proteins [37]. Li et al. discovered that T cell membrane proteins are transferred specifically to tumor target cells that present cognate HLA-I/peptide complexes. Taking advantage of these T cell-target cell interactions, they created a neoantigen discovery system by co-incubating T cells expressing a labeled orphan TCR with cognate target cells. With the fluorescent label being transferred from T cells to target cells, this method enabled the isolation of these target cells and sequencing of the cognate TCR ligand, thus establishing a library of NeoAgs [38]. Compared with pMHC yeast display [39], this method produces “orphan” TCR (i.e., TCRs of unknown antigen specificity) protein reagents, but instead uses orphan TCRs as expressed in their natural context.

### 3.4. Isolation of Tumor-Specific T Cells and T Cell Receptors (TCR)

Tumor-reactive T cells and TCRs can be identified from autologous, allogeneic, or xenogeneic repertoires, and the methods can use HLA-I multimers, single-cell TCR sequencing, or antigen-negative humanized mice [40,41,42,43]. With the HLA-I multimer method, antigen-specific CD8^+^ T cells can be directly isolated by multimer staining and flow cytometric sorting. These polyclonal T cells are then tested for cognate peptide recognition and antitumor function prior to proceeding to isolate paired full-length TCR sequences. This functional testing typically requires the expansion of T-cell populations, but non-expanded antigen-specific T cells can also be isolated in small numbers and subjected to a highly sensitive PCR-based single-cell TCR analysis method (TCR-SCAN). HLA-I multimer labeling and single cell-sorting with this method can yield TCRs with high affinity and specificity [44]. Humanized mice have also been generated to utilize a murine TCR repertoire that is not subjected to the same degree of T cell clonal deletion or tolerance as in humans. To this end, Li et al. generated transgenic mice with the entire human TCR alpha/beta gene loci and a chimeric HLA-A2 transgene containing a diverse T cell repertoire to enable the isolation of human TCRs against human TAAs [45].

Single-cell sequencing approaches represent much more promising methods to perform high-throughput isolation of tumor-specific TCR-encoding genes [40]. Using an RNA bait library that targets each individual variable (V) and joining (J) element within the TCRα and TCRβ loci allows the selective isolation of the TCR-encoding genome elements from sheared genomic DNA (gDNA) fragments for subsequent paired-end deep sequencing. This makes the identification of antigen-specific TCRs in oligoclonal T cell populations from either human material or TCR-humanized mice possible. Ton Schumacher’s group used this method to identify a large panel of TCRs derived from intratumoral CD8^+^ T cells, which were specific for shared tumor antigens [40]. This strategy also isolated novel TCRs that were reactive to yet-to-be-defined autologous tumor-associated antigens. This finding may help to exploit a broad, tumor-reactive repertoire of TCRs that is not limited by the current knowledge of the tumor immunopeptidome, which is an important first step toward the development of an autologous TCR gene therapy targeting patient-specific neoantigens. If tumor-specific TCRs can be rapidly re-introduced into autologous T cells, this would allow for ‘transplantation’ of the tumor-reactive TCR repertoire from exhausted T cells into a fitter T cell population [46].

Naïve T cells can also be a source of TCRs for TCR-T therapy. TAA- and NeoAg-specific T cells can be derived and expanded from low-frequency precursors in the peripheral blood of cancer patients and either re-infused or used as a source of antigen-specific TCRs. Since cancer patients often show immune suppression or dominant T cell tolerance, the naïve repertoire of HLA-I matched healthy donors also represents a reliable source due to its huge diverse TCR repertoire, which theoretically entails T cells for any antigen specificity, including tumor neoantigens. High-throughput mapping platforms have been developed to interrogate the naïve repertoire for fast and efficient identification of rare but therapeutically valuable TCRs for personalized adoptive T cell therapy [47].

## 4. TCR Cloning and Validation Approaches

Tumor antigen-specific TCR repertoires identified by next-generation sequencing can be used to genetically engineer T lymphocytes for TCR-T therapy. Most TCR-based gene therapy approaches rely on the ex vivo transduction of T cells with viral vectors. The first vectors used in gene therapy were adenoviruses [48]. However, since they are not able to integrate transgenes into the host genome, TCR expression was lost during T cell proliferation. Moreover, the immunogenetic features of adenoviruses also limit their use as gene therapy vectors. By contrast, retroviruses have shown much more promise as gene transfer vectors, since they can infect a wide variety of cells and have the capacity to insert transgenes into the host genome, which allows the ectopic TCR α/β chains to be stably expressed [49]. Retroviral vectors derived from gamma-retroviruses such as murine leukemia viruses (MLVs) have been widely used for gene transfer into human T cells. This method has been used to deliver various genes, including suicide genes, TCR genes, and CARs [1]. The major drawback is their inability to transduce non-proliferating target cells, which excludes the use of quiescent T cells in TCR-T therapy. In addition, potential side-effects may be caused by retroviral insertional mutagenesis, underscoring the importance of monitoring vector integration sites and developing safer vectors [50].

More recently, lentiviral vectors (LV) have gained more traction as gene transfer vectors, since they can deliver genes into both dividing and non-dividing cells. While the transduction of naïve T cells is ideal for TCR-T therapy aimed at providing long-lasting immune reconstitution, protocols have been developed to allow the efficient LV transduction of T cells in the absence of TCR triggering. The combination of IL-2, IL-7, and IL-15 promotes the long-term in vitro survival of memory and naïve T lymphocytes, and improves lentiviral transduction efficiency in the absence of TCR activation [51]. Various techniques, such as Golden Gate cloning and LR cloning are often used to construct the vectors for inserting the TCR α/β genes, and the recently introduced Gibson assembly appears to be very rapid and promising high-throughput method [52]. Adeno-associated virus (AAV) is another widely used viral vector [53,54]. Compared with adenoviral vectors, AAVs have lower immunogenicity and wider cell tropism, and thus have been widely applied in cancer gene therapy. To promote transgene integration, the self-complementary AAV vector (scAAV) was developed, which allowed AAVs to be independent from the host cell’s complementary strand synthesis [55]. Although this method lowered the vector packaging capacity, scAAV outperformed conventional AAVs in terms of efficacy in preclinical models [56,57].

Although they have been widely used, viral vectors do not insert target transgenes at a predetermined genomic location; therefore, they can potentially lead to the driving of native gene expression. Several strategies have been implemented to increase the safety profile of integrating vectors, such as the elimination of the viral genes responsible for virulence, splitting packaging genes into different plasmids, and the introduction of inactivation switches in the vector’s constructs. It is also feasible to insert whole viral genomes into genomic regions that are distant from important genes and DNA elements, thus minimizing the risk of perturbing the expression of native genes [58,59].

Meanwhile, several non-viral gene editing methods have been developed. mRNA electroporation has been proven to achieve transient TCR and CAR expression, thereby minimizing the risk of viral element persistence [60,61,62]. Clinical data have shown that both mRNA-modified TCR-T and CAR T cells are feasible and safe without overt evidence of off-target toxicity against normal tissues [61,62]. However, lack of persistent TCR expression may limit efficacy and necessitate repeated infusions.

The non-viral Sleeping Beauty retrotransposon system has also been used to successfully introduce TCRs and CARs into primary T lymphocytes by electroporation, although the attractive simplicity of this plasmid-based gene transfer system is somewhat offset by the reduced transduction efficiency compared with viral vectors, necessitating longer T-cell expansions prior to patient infusion [63,64].

Genome editing has brought the promise of specific and efficient insertions of large transgenes into target cells through homology-directed repair (HDR) [65,66]. Theodore Roth et al. developed a CRISPR-Cas9 genome targeting system that does not require viral vectors, allowing rapid and efficient insertion of large DNA sequences at predetermined sites in the genomes of primary human T cells while preserving cell viability and function. TCR-engineered T cells developed using this method have been shown to specifically recognize tumor antigens in vitro and induce productive anti-tumor responses in vivo [67]. Non-viral genome targeting accelerates the process from target antigen selection to production of genetically modified T cells and provides a lower-cost approach to re-programming T cells for the next generation of immunotherapies.

Following TCR cloning, extensive preclinical validations are necessary to demonstrate the specificity and safety of the engineered TCR-T cells, particularly when targeting shared TAAs. Validations include assessing the TCRs’ affinity by performing a titration of the cognate peptide antigen, as well as measuring the killing of a panel of HLA-I matched tumor cell lines. Target cells can be transduced to express the antigens and relevant HLA-I molecules if no such tumor cell lines exist. Safety testing includes testing the candidate TCR-Ts’ recognition of panels of HLA-I matched primary tissues to ensure that no normal tissues are targeted, which may lead to toxicities. Cross-reactivities against normal brain and heart cells have resulted in patient deaths in at least two clinical trials of TCR-T cell therapy [68,69,70]. However, it is important to note that one of the TCRs was generated in a transgenic mouse model and the other was modified to increase the TCR’s affinity for the target antigen, thus bypassing the natural tolerance mechanisms that ensure the deletion of dangerous self-antigen reactive TCRs in vivo. These trial results underscore the importance of extensive safety tests of TCRs prior to moving them into clinical trials, and sound a strong word of caution about the source of the TCRs used.

High-throughput approaches for screening hundreds of sequenced TCRs for tumor antigen recognition have now been developed, which will facilitate truly personalized TCR-T cell therapies. The antigen specificity of various cloned TCRs extracted from TIL and/or PBMC can be assessed by co-culturing them with Epstein-Barr virus-immortalized lymphoblastoid cell lines pulsed with hundreds of peptides corresponding to personalized neoantigens (NeoAgs), various TAAs, or common viral antigens such as HLA-I immunopeptidomes [71]. In a more unbiased approach, NeoAgs can also be expressed as mini-genes in autologous antigen-presenting cells to assess panels of TCRs for antigen reactivity [63,72]. The introduction of DNA libraries to facilitate the quick assembly of TCRαβ genes using a Sleeping Beauty system is another approach featuring tetramer-guided isolation of antigen-specific TCRs by paired single-cell TCRαβ cloning and the rapid assembly of TCRαβ genes [52,73]. Various reporter systems measuring luciferase activity or cytokine production utilize flow cytometry or fluorescence microscopy for signal detection. Many groups have used nuclear factor of activated T cell (NFAT)-inducible elements to generate reporter cell lines to conditionally measure antigen-specific activation through candidate TCRα/β pairs [52].

## 5. Clinical Trials of TCR-T Cell Therapy for Cancer

Among the 175 studies listed on ClinicalTrials.gov (accessed on 9 August 2021) using TCR-T therapies, 71 of them utilize specific TCRs targeting a defined TAA or neoantigen, and 32 studies have been completed. NY-ESO-1 is the most commonly targeted antigen, which is expressed by a wide range of cancers, including myeloma, melanoma, and multiple carcinomas (Table 1 and Table 2). Other cancer-testis antigens such as PRAME and MAGE proteins are also popular TCR-T targets, along with melanoma differentiation antigens MART-1 and gp100, and, more recently, cancer drivers such as WT1, KRAS, and TP53.

Eighty-three sponsor/collaborators are listed as participating in TCR-T cell therapies, including the National Institutes of Health (NIH), state government organizations, industries, and universities/academic institutions (Supplemental Figure S1). The National Cancer Institute (NCI) currently supports 53 projects, which constitute ~20% of all the ongoing trials. Among the 29 private companies developing TCR-T therapies, GlaxoSmithKline and Adaptimmune have initiated the most clinical trials, with 11 trials and 7 trials listed, respectively. More recently, a Phase 1 clinical trial of TCR-T cells engineered with a TCR targeting the human papillomavirus (HPV)-16 E7 protein for the treatment of metastatic human papillomavirus-associated epithelial cancers was reported (NCT02858310). In this study, robust tumor regressions were observed with objective clinical responses reported in 6 of 12 treated patients [74]. This represents a milestone clinical trial for TCR-T cell therapies, demonstrating that targeting viral antigens showed effective clinical results for patients with viral-associated cancers. Other viral antigens being explored as TCR targets include HPV-E6 proteins, antigens derived from Epstein-Barr virus (EBV), and human endogenous retroviral (HERV) targets such as HERV-E (Table 1).

TCR-T therapies targeting the TAAs MART-1 and NY-ESO-1 have also demonstrated clinical efficacy in advanced melanoma, myeloma, and non-small cell lung cancer (Table 2). The overall response rates (ORR) of completed TCR-T clinical trials ranged from 0% to ~60%, but almost half of the trials did not report clinical response rates. It is worth noting that most of these TCR-T clinical trials enrolled small populations of patients (2 to 25), and therefore the ORRs are not likely to be statistically accurate. Therefore, larger Phase II and III clinical trials are needed to confirm actual clinical efficacy of these TCR-T therapies. A number of different factors have contributed to the lack of consistent clinical success: (1) immune toxicities caused by targeting of normal tissues, (2) insufficient or transient TCR expression in engineered T cells, (3) T cell exhaustion and dysfunction, (4) tumor immune escape, and (5) lack of validated tumor-specific antigens to target in most cancer patients. Overcoming these challenges will be critical for achieving greater clinical success in the future.

## 6. Challenges and Potential Solutions for Improving TCR-T Cell Therapy

**Figure 3:**
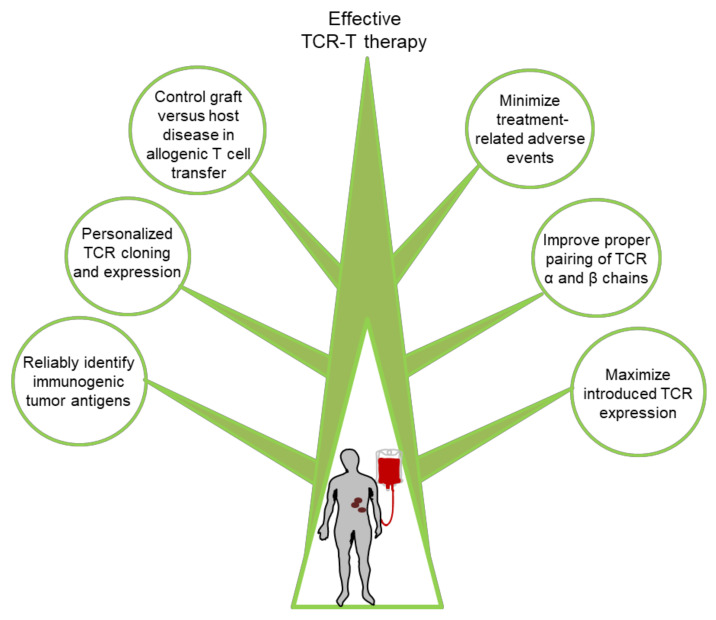
Summary of approaches that can improve TCR-T cell immunotherapy.

Although TCR-T cell-based immunotherapies have demonstrated clinical efficacy in a substantial subset of treated patients, improvements are still needed in a number of areas as discussed further below (Figure 3).

### 6.1. Discovery of New Targets

There is currently a very limited set of peptide antigen targets for facilitating effective and safe TCR-T based immunotherapies. Most of the currently used targets are TAAs that although upregulated in tumor tissue still maintain low levels of expression in normal tissues, which may lead to autoimmune toxicities or to tolerance of the engineered T cells. Therefore, neoantigens appear to be the safest targets for TCR-T cancer therapies [75,76]. However, major challenges for the clinical exploitation of neoantigens in TCR-mediated ACT include the following: (1) neoantigen-forming mutations are largely private and differ between cancer patients, making it difficult to develop a widely applicable immunotherapeutic product; and (2) the expression of NeoAgs is frequently heterogeneous across tumor tissues [77]. Despite this, reports in recent years have highlighted the occurrence of immunogenic neoantigens that are widely shared by tumor cells, including mutated KRAS and TP53 [78]. Our group has reported a neoantigen peptide derived from the EGFR-L858R mutation (KITDFGRAK) that is immunogenic and presented by HLA-A*1101. Since EGFR-L858R is found in ~40% of EGFR-mutated lung cancers, it constitutes a promising, widely shared NeoAg target for TCR-T therapy of HLA-A*1101/EGFR-L858R lung cancer patients [28]. Our group has generated several promising TCRs specific for this and other EGFR-derived NeoAgs, and we plan to test their efficacy in future clinical trials [28,29]). A number of other studies have also demonstrated the immunogenicity of shared NeoAgs that can be used to generate potentially therapeutic tumor specific TCRs [79,80,81]. With the recent advances in next-generation sequencing technology, particularly single-cell DNA sequencing, transcriptome sequencing, and well-developed in vitro validation approaches, including ELISPOT assay and tetramer staining, it is possible that TCR-T immunotherapy targeting personalized neoantigens will become a feasible approach for cancer treatment in the years to come. Furthermore, new categories of emerging TAAs such as cancer-placenta antigens may also constitute viable shared targets for future TCR-T development [27].

### 6.2. Maximizing Therapeutic TCR Expression

The proper pairing of transgene α and β chains is one of the central challenges encumbering the development of TCR-T cells [82,83]. Since each transduced T cell includes two endogenous TCR chains and two transformed TCR chains, heterodimers with unknown specificity can lead to potential autoimmune consequences, which has been demonstrated to occur in some mouse models [84]. Another related concern is that inappropriate α/β chain TCR pairs will compete for the CD3 complex, thus lowering the surface expression and signal transduction of the therapeutic TCR [85]. There are several methods that favor appropriate pairing of the transduced TCR chains by engineering, including: (1) partial murinization of the TCR’s constant regions [86,87], (2) the addition of cysteine residues to promote disulfide bonding of the introduced TCR chains [88,89,90,91], (3) altering the secondary structure of the endogenous TCR’s constant regions [92], (4) adding signaling domains to the intracellular portions of transduced TCRs [92], and (5) introducing TCR-α/β chains into alternative effector cells [93,94] or constructing single chain TCRs [82]. Methods of enhancing therapeutic TCR expression include: (1) codon optimization of TCR-α and TCR-β chain transgenes [95,96], and (2) altered TCR-α/TCR-β vector configurations to optimize expression [97].

### 6.3. Minimization of Adverse Events

Although TCR-T cell therapies have shown remarkable clinical responses, adverse events have been documented. As mentioned above, high-affinity TCRs targeting MART-1 and gp100melanoma antigens induced severe histological destruction in normal tissues where melanocytic cells were present, including the skin, heart, eyes, and inner ears [68,69,70,98]. Generally, on-target off-tumor toxicities represent the major critical obstacle for TAAs that are shared by healthy tissues or that cross-react against structurally similar epitopes [99]. However, other melanoma differentiation antigens such as SLC45A2 show much lower levels of expression in normal tissues, which may reduce the risk of autoimmune side-effects. However, this risk has provided a motivation for researchers to investigate the shared neoantigens more closely [100,101]. Currently, multiple oncogene hotspot mutations are being investigated as potential TCR targets, such as phospatidylinositol-3-kinase (PI3K), KRAS and TP53, or the breakpoint regions of oncogenic fusion proteins [102,103]. In light of previous experience, post-infusion monitoring is essential, and the ability to delete infused TCR-T cells through genetic engineering with suicide genes is an important safety measure to employ [104]. Clearly, developing the ability to reliably identify personalized, highly specific and immunogenic tumor antigen targets will be crucial for minimizing the adverse events associated with TCR-T cell therapies [105].

### 6.4. Graft Versus Host Disease in Allogenic T Cell Transfer

The use of allogeneic T cells is a highly promising solution to overcome manufacturing issues, patient-related immune cell defects, and delays in treatment. For proper use of allogeneic T cells, it is necessary to control the issues of graft-versus-host disease caused by transduced alloreactive lymphocytes and rejection of engineered lymphocytes by the host’s immune system [106,107]. The deletion of endogenous TCR genes, HLA-I loci, or the CD52 molecule are among the strategies for avoiding TCR-T graft failure, which can be achieved by several methods such as gene editing or using siRNA [107,108,109,110]. Pluripotent stem cell technology has also been suggested as a potential solution [111]. Meanwhile, the impact of TCR disruption on T cell proliferation and survival is still not fully understood [112]. Therefore, more preclinical and clinical data are needed to understand and navigate these issues.

## 7. Conclusions

TCR-T therapies represent a highly promising immunotherapeutic modality for cancer treatment. Increasing numbers of studies in this field are beginning to shed light on the discovery and cloning of personalized, tumor antigen-specific TCRs and the cutting-edge techniques involved. However, improving the anti-tumor efficacy of TCR-T immunotherapy still has several key challenges, including how to safely increase the avidity of therapeutic TCRs, how to identify shared tumor-specific antigens and TCRs in a given patient population, how to utilize personalized TCRs in cancer patients; and what interactions or signals regulate TCR expression and optimal function. Future studies that decipher these and related questions will not only enable a better fundamental understanding of TCR-T therapy, but will also provide as yet untapped opportunities for improved TCR-T treatment efficacy aimed at increasing the overall survival of cancer patients.

## Figures and Tables

**Table 1:** Tumor antigens currently targeted by TCR-T cells.

Antigen	Number of Studies	Cancer Type	Trial Registration Number (ClinicalTrials.gov (Accessed on 9 August 2021))
HPV16-E7	6	Neoplasia Oropharyngeal neoplasms	NCT04476251 (suspended) NCT04411134(withdrawn) NCT04044950 (withdrawn) NCT04015336 (suspended) NCT02858310 (recruiting) NCT03937791 (terminated)
HPV16-E6	3	Cervical cancer Head and neck squamous cell carcinoma Vaginal cancer Anal cancer	NCT03578406 (recruiting) NCT02280811 (completed) NCT03197025 (terminated)
KRAS	3	Pancreatic cancer Pancreatic ductal adenocarcinoma Gastric cancer Gastrointestinal cancer	NCT04146298 (recruiting) NCT03190941 (suspended) NCT03745326 (suspended)
MAGE	10	Melanoma, uveal Head and neck cancer Metastatic lung non-small cell carcinoma Breast cancer Metastatic malignant solid neoplasm Urinary bladder cancer Cervical cancer Renal cancer	NCT04729543 (recruiting, MAGE-C2) NCT04639245 (recruiting, MAGE-A1) NCT02153905 (terminated, MAGE-A3) NCT02111850 (complete, MAGE-A3) NCT03139370(recruiting, MAGE-A3/A6) NCT02096614 (complete, MAGE-A4) NCT03132922 (recruiting, MAGE-A4) NCT03247309 (recruiting, MAGE-A4/8) NCT03441100 (recruiting, MAGE-A1) NCT02989064 (complete, MAGE-A10)
LMP2	2	Nasopharyngeal carcinoma	NCT03925896 (recruiting) NCT04509726 (not yet recruiting)
TAC-1	1	HER2-positive solid tumors	NCT04727151 (recruiting)
MLANA (MART-1)	5	Melanoma Skin cancer	NCT00612222 (terminated) NCT00509288 (complete) NCT00923195 (complete) NCT00706992 (terminated) NCT00910650 (complete)
EBV	4	Nasopharyngeal carcinoma Head and neck squamous cell carcinoma Acute myeloid leukemia Neuroblastoma	NCT03648697 (recruiting) NCT04139057 (recruiting) NCT00085930 (active, not recruiting) NCT01430390 (recruiting)
PMEL(gp100)	3	Skin cancer Melanoma	NCT00509496 (terminated) NCT00923195 (complete) NCT03649529 (recruiting)
HERV-E	1	Kidney cancer	NCT03354390 (recruiting)
HA-1H	2	Juvenile myelomonocytic leukemia Recurrent acute biphenotypic leukemia Recurrent acute undifferentiated leukemia	NCT04464889 (active, not recruiting) NCT03326921 (recruiting)
TP53	2	Kidney cancer Melanoma (skin)	NCT00393029 (complete) NCT00704938 (terminated)
WT1	5	Myelodysplastic syndromes (MDS) Acute myeloid leukemia (AML) Advanced pleural malignant Chronic myeloid leukemia Recurrent non-small cell lung carcinoma	NCT02408016 (active, not recruiting) NCT02770820 (active, not recruiting) NCT02550535 (complete) NCT01621724 (complete) NCT01640301 (active, not recruiting)
PRAME	1	Acute myeloid leukemia Myelodysplastic syndrome Uveal melanoma	NCT02743611 (active, not recruiting)
AFP	4	Hepatocellular carcinoma	NCT03132792 (recruiting) NCT03971747 (recruiting) NCT04665388 (recruiting) NCT04368182 (recruiting)
CTAG1A (NY-ESO-1)	26	Lung cancer Ovarian carcinoma Fallopian tube carcinoma Primary peritoneal carcinoma Liver cancer Gastric cancer Esophageal cancer Neoplasms Myeloma Advanced melanoma Bladder carcinoma Breast cancer Esophagus carcinoma Synovial sarcoma	NCT04878484 (not yet recruiting) NCT02775292 (complete) NCT03240861 (recruiting) NCT01967823 (complete) NCT00670748 (terminated) NCT01697527 (active, not recruiting) NCT01567891 (complete) NCT03691376 (active, not recruiting) NCT01457131 (terminated) NCT02869217 (recruiting) NCT02062359 (terminated) NCT03159585 (complete) NCT02070406 (terminated) NCT02588612 (complete) NCT03709706 (recruiting) NCT04526509 (recruiting) NCT03017131 (active, not recruiting) NCT01892293 (terminated) NCT03462316 (recruiting) NCT01352286 (complete) NCT04318964 (recruiting) NCT01350401 (terminated) NCT03250325 (active, not recruiting) NCT03168438 (terminated) NCT02992743 (active, not recruiting) NCT03399448 (terminated)

HPV-E7, human papillomavirus 16 (E7); HPV-E6, human papillomavirus 16 (E6); KRAS, Kirsten rat sarcoma virus; MAGE, melanoma-associated antigen; LMP2, latent membrane protein 2; TAC, protachykinin-1; MART-1, melanoma antigen recognized by T cells-1; EBV, Epstein–Barr virus; gp100, glycoprotein 100; HERV-E, human endogenous retrovirus group E; HA-1H, a 29-mer peptide derived from histocompatibility antigen; TP53, tumor protein 53; WT1, Wilm’s Tumor-1 transcription factor; PRAME, preferentially expressed antigen in melanoma; AFP, alpha-fetoprotein; NY-ESO-1, New York esophageal squamous cell carcinoma-1.

**Table 2:** Reported TCR-T clinical trials for cancer.

Trial Registration Number (ClinicalTrials.gov (Accessed on 9 August 2021))	Target Antigen	Cancer Type	Trial Stage	Clinical Response Results	Year
NCT00509288	MART-1	Metastatic melanoma	Phase 2	PR 6/21	2012
NCT00923195	MART-1 Gp100	Melanoma, skin cancer	Phase 2	CR 0/2 PR 0/2	2015
NCT02280811	HPV16-E6	Vaginal, cervical, anal, penile, oropharyngeal	Phase 1 and 2	PR 2/6	2017
NCT01352286	NYESO	Multiple myeloma	Phase 2	CR 3/25 PR 18/25	2019
NCT01967823	NYESO	Melanoma, meningioma, beast, non-small cell lung, hepatocellular	Phase 2	CR 10% PR 50%	2021
NCT02858310	HPV16-E7	Virus-associated epithelial cancers	Phase 1	PR 6/12	2021

HPV-E7, human papillomavirus 16 (E7); HPV-E6, human papillomavirus 16 (E6); MART-1, melanoma antigen recognized by T cells-1; Gp100, glycoprotein 100; NY-ESO-1, New York esophageal squamous cell carcinoma-1.

## Data Availability

Not applicable.

## Supplementary Materials

The following are available online at https://www.mdpi.com/article/10.3390/cells10092379/s1. Figure S1: Academic sponsor/collaborators that working on TCR-T therapy and private companies involved in TCR-T therapy.
