# Supplementary material for: Evolution of CD8+ T Cell Receptor (TCR) Engineered Therapies for the Treatment of Cancer
Source: Cells. 2021 Sep 10;10(9):2379. doi: 10.3390/cells10092379 (PMC8469972; doi:10.3390/cells10092379)
Supplement: Supplementary file 1 [file cells-10-02379-s001.zip › cells-1353423-supplementary.pdf]

A.

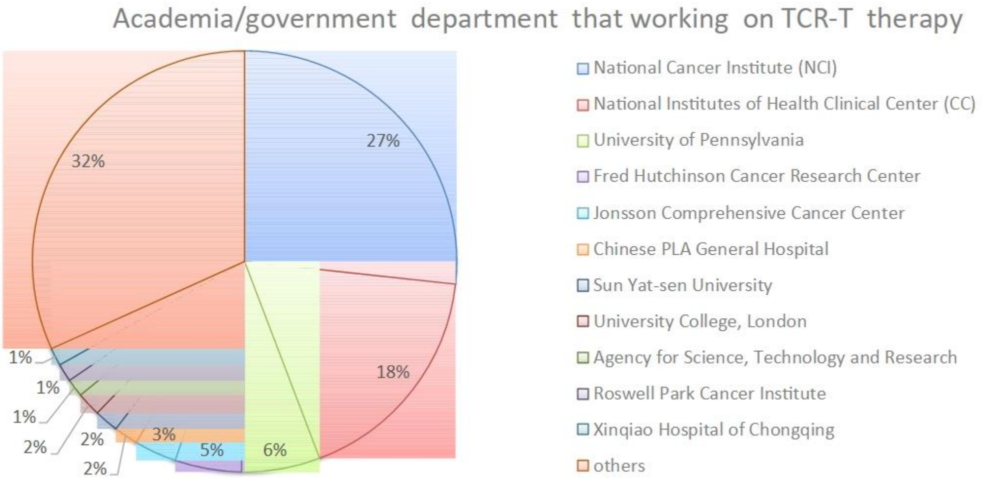


SPPLEMENTAL FIGURE 1

B.

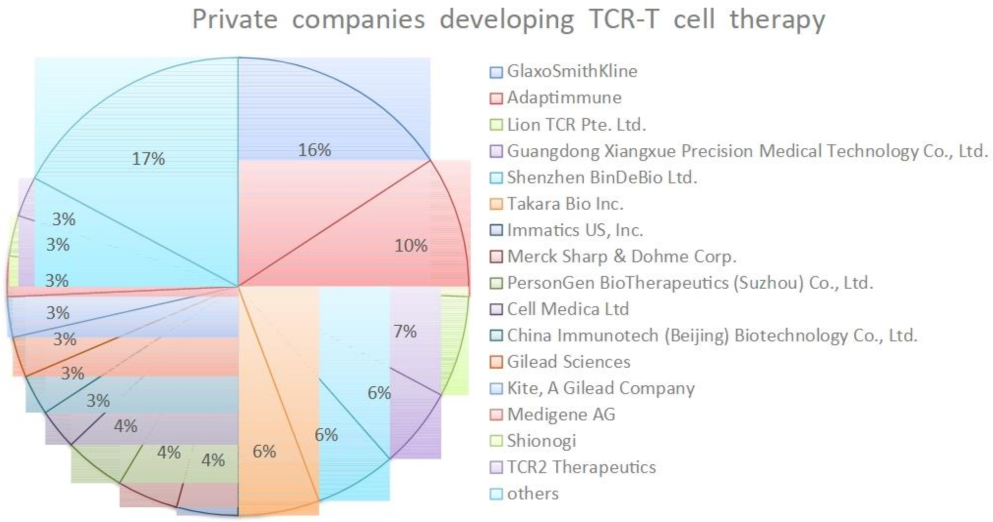


SPPLEMENTAL FIGURE 1

**Supplemental Fig.1** Academic sponsor/collaborators that working on TCR-T therapy(**A**), and private companies involved in TCR-T therapy (**B**). Percentages represents the proportion of numbers of TCR-T clinical trials at each institution.
